# A novel single cell method to identify the genetic composition at a single nuclear body

**DOI:** 10.1038/srep29191

**Published:** 2016-07-08

**Authors:** David Anchel, Reagan W. Ching, Rachel Cotton, Ren Li, David P. Bazett-Jones

**Affiliations:** 1 Genetics and Genome Biology Program, The Hospital for Sick Children, Toronto M5G 0A4, Canada; 2 Max Planck Institute of Immunobiology and Epigenetics, Department of Epigenetics, Freiburg 79108, Germany

## Abstract

Gene loci make specific associations with compartments of the nucleus (e.g. the nuclear envelope, nucleolus, and transcription factories) and this association may determine or reflect a mechanism of genetic control. With current methods, it is not possible to identify sets of genes that converge to form a “gene hub” as there is a reliance on loci-specific probes, or immunoprecipitation of a particular protein from bulk cells. We introduce a method that will allow for the identification of loci contained within the vicinity of a *single* nuclear body in a single cell. For the first time, we demonstrate that the DNA sequences originating from a *single* sub-nuclear structure in a single cell targeted by two-photon irradiation can be determined, and mapped to a particular locus. Its application to single PML nuclear bodies reveals ontologically related loci that frequently associate with each other and with PML bodies in a population of cells, and a possible nuclear body targeting role for specific transcription factor binding sites.

A prevailing theme of nuclear organization is that processes of genomic regulation are both imparted by, and contribute to, the compartmentalization of nuclear factors into enriched foci called “nuclear bodies” (NBs). The observation of specific genomic/nuclear body associations, including U2 gene loci at Cajal bodies^1^, Hemaglobin b (Hbb) and other erythroid specific genes at splicing speckles^2^, and the p53 locus at PML bodies^3^, has led to the idea of the nuclear body as a “gene hub”, whereby the co-regulation of sets of genes, either in *trans*, or separated by large regions on the same chromosome, is correlated with their shared association with a nuclear body enriched for their cognate regulatory factors^4^,^5^,^6^.

Crucial to the testing of the “gene hub” model is the ability to detect multiple loci that are convergent at a *shared* nuclear body. Their detection would also provide insight into the function of the associated body, and may reveal novel co-regulatory states that precede a change in transcription and are thus undetectable by conventional gene profiling approaches^7^. So far, loci/nuclear body associations have been revealed by population-based approaches that rely on the cross-linking of DNA with either a constituent protein of the body (i.e. ChIP;)^8^ or an hapten deposited in its vicinity (i.e. immuno-Trap)^9^ that is precipitated in a biochemical pulldown, or by a direct observation of the nuclear location of an *in situ* labelled candidate genomic locus (i.e. immuno FISH)^10^. However, immuno-FISH is intended for the determination of the spatial relationship of a selected locus of interest with the nuclear landscape and is not feasible for detecting novel loci/protein associations. Moreover, presently available biochemical approaches are not feasible if the starting material is limiting (e.g. embryos)^11^ or the interaction is transient^12^. Biochemical methods would also not distinguish between nuclear bodies differing in morphology, subnuclear location, or heterogeneity in composition of specific factors within single cells^12^,^13^,^14^. These established methods would also not be suited to nuclear bodies in which the constituent factor that binds to chromatin is not known^15^, or if solubility becomes problematic during isolation and purification steps^9^. In particular, the current techniques cannot identify associations with NBs that have unique morphology, composition of factors, a specific subnuclear location, or are otherwise biochemically indistinguishable from other structures within the same cell.

In the acute promyelocytic leukemia derived NB4 cell line, there are a fusion protein created by a translocation involving the PML and Retinoic Acid Receptor Alpha genes (PML-RARα) results in a differentiation block of myeloid progenitor cells, and a disregulation of RARα target genes, that is concomitant with a dispersal of PML NBS into smaller, more numerous “micro - PML” accumulations, which are no longer associated with typical PML body components such as CBP and SP100. While the majority of these “micro -PML” foci are representative of the PML-RARα fusion protein at RARα target genes^14^,^16^,^17^, our lab has observed that in some cells there are one to three PML bodies that remain intact by virtue of their larger size, and presence of PML body constituent proteins (i.e. SP100)^14^. If the integrity of PML bodies are lost in the NB4 cell line due to the association of the PML-RARα fusion protein with RARα target genes, we are interested in possibility that these relatively intact bodies represent a “gene hub”, whereby PML body components are retained through particular specific genomic contacts. As explained above, ChIP would not be suitable to elucidate the genomic contacts surrounding these “unique” PML bodies since the precipitate would also contain genomic DNA in contact with the micro-PML accumulations. To determine the loci convergent at these particular bodies, and as a general method for the dissection of sub-nuclear structures that are presently intractable, we were motivated to develop a novel approach that allows for the dissection of the genomic neighbourhood of a *single* nuclear body.

To this end, we report here a novel technique called laser targeted oligo ligation (LTOL). It is based on the amplification and sequencing of DNA at sites of double strand breaks (DSBs) induced by targeted 2-photon irradiation focussed at a sub-nuclear structure of interest. We demonstrate that this approach is sufficiently sensitive to yield DNA sequences originating from the targeted region of single nuclear bodies, and of sufficient length to be mapped to a specific genomic locus. As proof-of-principle, we targeted single histone locus bodies (HLBs), and show that the resultant sequences are enriched for those that cluster near the expected colocalizing histone gene locus^18^. Our targeting also revealed a novel locus at HLBs which we confirmed by the immuno-FISH method. We then applied the LTOL technique to those PML bodies found in the NB4 APL cell model^14^ that retain their constituent proteins. We found two DNA sequences that originate from the targeting of a single PML body. Distance measurements of FISH signals corresponding to these loci show that they frequently co-localize with each other and with PML bodies in a population. This result underscores the value of our technique in the detection of convergent loci at a shared nuclear body. By combining information of loci identified at PML NBs by LTOL and our previously described targeting approach (i.e. ImmunoTrap)^9^, we observe the prevalence of particular transcription factor binding sites (e.g. SP1) may have a role in the targeting of specific loci to PML NBs. Although the technique is at present limited in the number of sequences obtained with each experiment, the false positive rate (i.e. sequences that do not associate by FISH in a cell population with the type of body targeted) is sufficiently low that it can still be used for uncovering genuine novel genomic/nuclear body associations, including loci from different chromosomes that are associated at a shared nuclear body. Our report introduces LTOL as both a technological benchmark for the determination of DNA sequences in arbitrary volumes at single sub-nuclear structures *in-situ*, and as an application to find loci/nuclear body associations in those bodies that cannot be dissected by conventional biochemical assays.

## Results

### Single molecule amplification and sequencing of DNA originating from a single nuclear body

**Figure 1:**
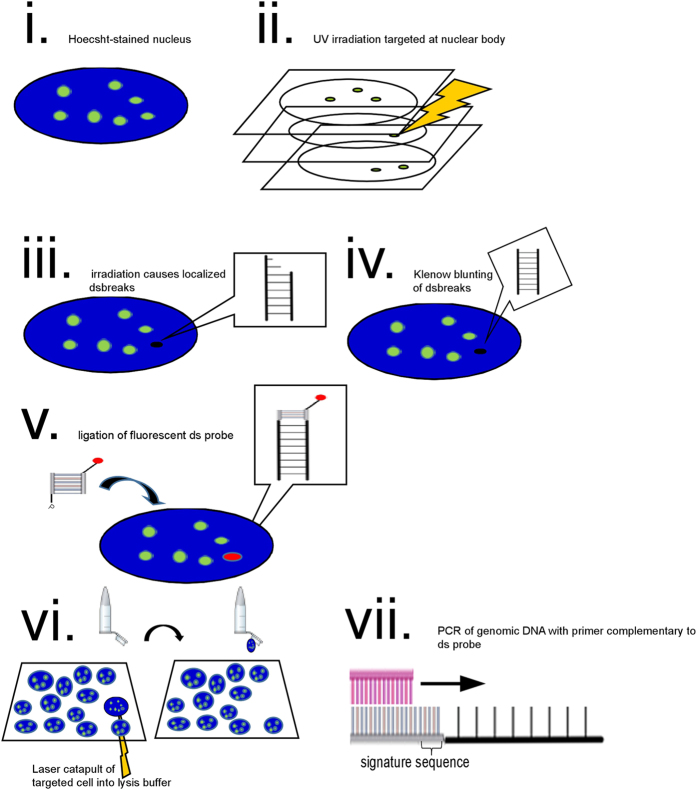
(**a**) i. Cells contained within an etched parallelogram (a “keystone”) on a coverslip are immunolabelled and Hoechst stained. ii. In the fluorescent channel of the immunolabelled signal, a Z-stack is recorded. A 2-D region of interest confined to a chosen number of stacks is targeted with two-photon irradiation. This bleaches the Hoechst in the targeted volume, causing localized DNA double strand breaks (dsbs). iii. To blunt the DNA for ligation with the blunt end probe, the cells are incubated with Klenow enzyme and DNTPs. iv. The cells are then incubated with a blunt end oligo and T4 DNA Ligase. The oligo contains priming sites that are used for the subsequent amplification steps. To prevent intra-probe ligation the oligo lacks 3′OH groups. Ligation to blunted genomic DNA occurs between a 5′phosphate contained on one strand of the oligo (binding strand) and a 3′hydroxyl of the blunted genomic DSB. (**b**) Single targeted cells (red arrowheads) are located the keystone for LMPC (Laser Micro-dissection Pressure Catapulting) isolation into lysis buffer. (**c**) The lysed cell is then subjected to PCR amplification with primers complementary to probe, to sufficient amplicon yield for sequencing. Primers used for PCR are 4bp shorter than the probe sequence so that amplicons that occur by mispriming events can be discounted by the lack of the “signature sequence” immediately following the primer sequence (see Supplementary Figure S1).

Our approach rests on *In Situ* Oligo Ligation (ISOL), originally developed for the *in situ* detection of DNA damage resulting from apoptosis in tissue preparations or cell culture^19^. It involves the ligation of double stranded oligo probes (with only one free 3′OH end to prevent intra-probe ligation) to DNA double strand breaks (DSBs) and subsequent detection via fluorescence microscopy or immunohistological staining. Because of its original use in studies of apoptosis, ISOL was developed for the detection of grossly induced DSBs, either by apoptotic or direct DNA damaging agents (e.g. hydrogen peroxide and DNase treatment respectively)^20^,^21^. However, we have found that it is possible to ligate blunt double stranded probes to submicron subnuclear regions induced by targeted 2-photon irradiation in the presence of a photosensitizer (i.e. Hoechst 32258)^22^. This is the basis of our approach, which we call Laser Targeted Oligo Ligation (LTOL) (see Fig. 1). Briefly, probes are ligated to Klenow-blunted DSBs induced at submicron subnuclear regions by targeted 2-photon irradiation (Fig. 1a). The targeted cell is then isolated in lysis buffer by a laser-catapulting microscope^23^ (Fig. 1b), and subjected to PCR using primers complementary to the ligated strand of the probe (Fig. 1c). Thus, DNA in those targeted subnuclear regions are specifically amplified to sufficient yields for their cloning and sequencing. The resultant sequences are screened for mispriming artifacts by the presence of the entire probe template sequence, including a 4bp “signature” sequence that is not found on the primer (see Materials and Methods, Supplementary Figure S1, and Fig. 1). For those sequences that map unambiguously to a genomic locus, fluorescent BACs corresponding to those loci can then be used in an immuno-FISH assay in order to verify if their nuclear body association is significantly frequent in a cell population. In this way, novel loci associations can be detected at arbitrary subnuclear regions in *single* cells, and without the need for an immunoprecipitation step. This allows for the genomic dissection of those nuclear bodies that, so far, are intractable to conventional biochemical/population-based methods (see above). Furthermore, because LTOL allows for the determination of DNA in the vicinity of a *single* nuclear body, it is ideally suited for the detection of novel multi-loci convergences that are predicted by the gene hub model.

**Figure 2:**
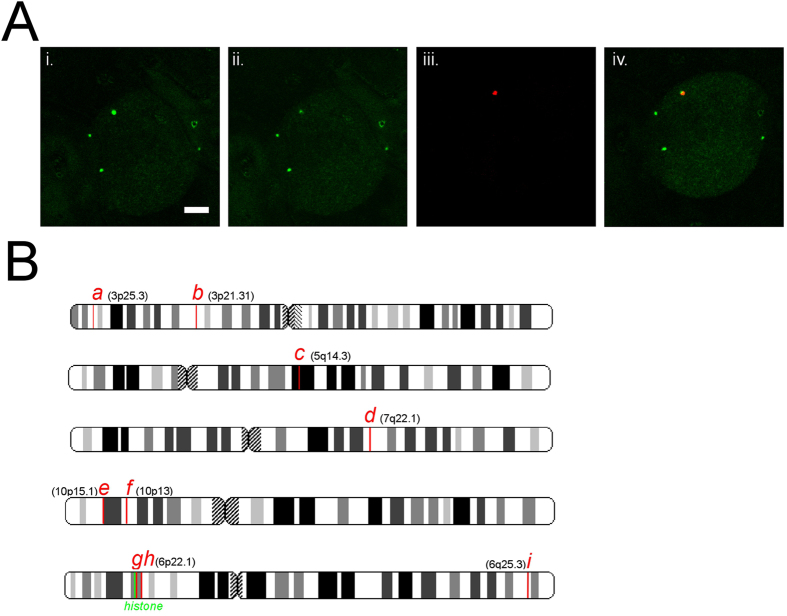
(**A**) Laser targeting of single HLB body. i. An HLB body (green foci) is targeted by two-photon irradiation (see Materials and Methods). ii. After irradiation the immunofluoresence of a single HLB body is bleached (compare green foci between i. and ii.). iii. After targeting a single HLB body and ligating an oligo (see Materials and Methods), an enriched focus of fluorescent oligo (red) can be seen. iv. The enriched fluorescence from the ligated oligo colocalizes with the targeted region. (**B**) chromosomal locations of sequences obtained from targeting of single HLBs (indicated by red lines). Two sequences were obtained that clustered to within 2 MB of the histone gene cluster (histone gene cluster is indicated by green line at 6p22.1). Annotation of genomic regions covered by LTOL sequences on chromosome 10 and those on chromosome 6 in the vicinity of the histone cluster can be seen here: (chromosome 6 region) and (chromosome 10 region).

As proof-of-principle of the LTOL method, we set out to determine if it is possible to target and determine the sequence of chromatin in the vicinity of a single sub-nuclear structure. An ideal candidate is the Histone Locus body (HLB), so-called because of its frequent colocalization with the histone clusters found at chromosomes 6p22.1 and 1q21.2 (Fig. 2)^18^. We immunolabelled HLBs in HT1080 cells with an antibody to the HLB’s constituent NPAT protein^24^, and induced DSBs in the vicinity of a single HLB by targeted 2-photon irradiation (see Fig. 2 and Materials and Methods). Subsequent labelling of targeted cells with fluorescent oligomers shows a clear enrichment of signal that overlaps with the NPAT signal, indicating the specific ligation of oligomers to chromatin in the vicinity of the HLB (Fig. 2). Although the spot size can be increased with increased laser power, we were able to confine the induced damage to approximately 600 nm radially, and 1 μm axially (Supplementary Figure S1). Single targeted cells were laser catapulted into individual microcentrifuge tubes, and the DNA amplified in parallel using primers complementary to the oligo probe sequence (see Materials and Methods). Parallel amplification of DNA obtained from targeting of single HLB bodies in 9 cells resulted in 9 sequences with a flanked “signature” sequence that could be unambiguously mapped to a specific genomic locus. The sequences were both significantly enriched for chromosome 6 (3 out of 9 sequences; p < 0.01 by the binomial test, see Materials and Methods), and clustered to within 1 and 2 Mb of the histone gene locus at 6p22.2 (2 out of 9 sequences; p < 7 × 10^−5^). As negative controls, cells that were not targeted, as well as cells that were targeted at regions not containing an HLB, were processed, and only yielded sequences that did not contain a flanked signature sequence (i.e. mispriming artifacts; see Supplementary Figure S1). These results indicate that the laser targeting technique is sufficiently sensitive to obtain sequences originating from the targeting of a single nuclear body, and furthermore, that the targeting is sufficiently accurate to yield sequences that cluster at the specific sub-chromosomal regions that colocalize with the nuclear body.

### FISHing of loci obtained from HLB targeting reveals a novel HLB-locus association

**Figure 3:**
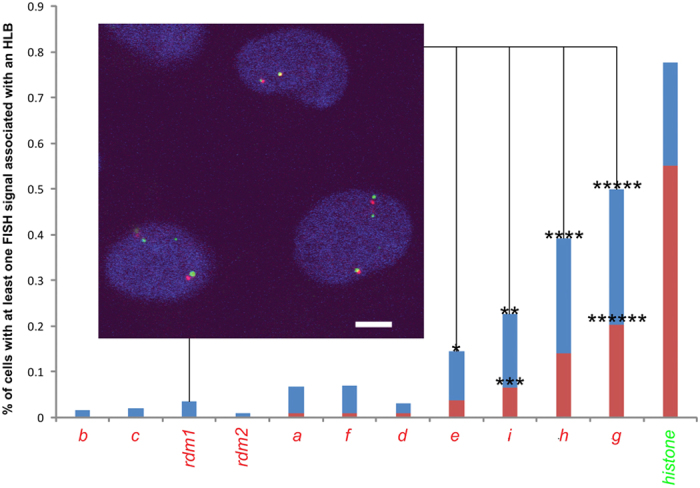
Measurements of the frequency of association (as indicated by red:600 nm and blue:1 μm centre-to-centre distances from an HLB) of sequences obtained from HLB targeting. Also shown are the frequency of association of two random BACs (indicated by *rdm1* and *rdm2*). X-axis labels correspond to BACs overlapping with sequence at chromosomal location indicated in Fig. 2(B). The green “histone” column refers to a BAC overlapping with the histone gene cluster at chromosome 6p22.1. See Supplementary Table S1 for listing of BACs used. (*p < 0.016; **p < 0.03; ***p < 0.0016; ****p < 0.0007; *****p < 4e^−9^; ******p < 0.000008; *******p < 1.3e^−11^). Inset: FISH of BAC corresponding to locus at *g* (red) with HLBs (green).

Using BACs overlapping with the genomic coordinates of the obtained sequences, we performed immuno-FISH to verify whether they indeed colocalized with HLBs in a cell population. As expected, the two sequences obtained within 1 MB and 2 MB from the histone locus clusters on 6p22.2 colocalized with significant frequency with HLBs (see Fig. 3). Furthermore, although the more distant loci captured on chromosome 6 at 6q25.3 (“*I*” in Fig. 3) significantly associated with HLBs, it appears the association is less frequent compared with those loci clustering at 6p22.2. Thus, the thresholding distances used in this analysis are sufficiently small to identify those sub-chromosomal regions that specifically associate with a targeted nuclear body. Using BACs, we also tested sequences obtained from targeting that did not map to chromosome 6, to see if these were false positives or did indeed associate with HLBs. Surprisingly, one sequence, mapping to within 10 kb of the RBM17 gene on chromosome 10p15.1, and originating from a targeting that also yielded a sequence proximal to the histone locus (Fig. 2B), significantly localizes in a cell population to within 1 μm of an HLB compared with random BAC controls (p < 0.016; See Materials and Methods- 3-D FISH and Imaging). Although it is unclear whether this frequent association is due to this particular locus on chromosome 10, it is at least noteworthy that the protein product of RBM17, SPF45, plays a role in RNA splicing associated with Cajal bodies^25^, which frequently localize adjacent to HLBs. Whether or not there is a functional relationship between the location of the gene locus and the gene product’s site of action remains to be tested. Regardless, this initial finding underscores the power of the LTOL technique; we have detected a novel, significant association of a gene locus with a nuclear body that could not be identified with existing approaches.

### Identification of paired loci that associate with PML bodies

The ability to identify genetic loci that localize in the vicinity of a *single* nuclear body is particularly suited to the interrogation of genomic/nuclear body associations that are not amenable by population-based biochemical approaches. This can occur when the nuclear body in question is “unique” in some way from other bodies, such as differing in compositional properties (e.g. concentration of a particular transcription factor). As explained above, only between 1 and 3 PML bodies are found in the acute promyelocytic leukemia-derived NB4 cell line, in a background of numerous micro-PML foci. The small foci presumably arise from the dispersal of normal PML bodies when the fusion transgene is expressed^14^,^16^,^17^. The LTOL technique then, is particularly suited to interrogate those particular PML bodies in the acute promyelocytic leukemia-derived NB4 cell line that retain their size and compositional integrity, since they may prevail because of particularly strong affinities with specific, convergent genomic contacts. We obtained several *bona fide* sequences (i.e. containing flanking “signature” sequence - see above and Materials and Methods) from the targeting of single intact PML bodies (i.e. containing both SP100 and PML) that mapped unambiguously to genomic loci (Fig. 6C). To determine whether any of these regions are specifically associated with these intact bodies in a population of NB4 cells, we performed immuno-FISH using fluorescently labeled BACS that overlapped with these sequences, and measured their frequency of association with intact PML bodies. We observed a significant association of two BACs with PML bodies, mapping to p11.2 and q13.31 of chromosomes 17 and 20 respectively (Fig. 6D). These two sequences originated from the targeting of a single PML body, raising the possibility that their convergence at a shared PML body may be more frequent than that predicted by considering their independent association. Indeed, paired FISH of these two loci revealed that they significantly co-localized with each other compared with the random controls (Fig. 7A), and although their colocalization could occur in the absence of a common PML body, the chromosome 20 locus was significantly closer to a PML body when a chromosome 17 locus was within 1 μm of the body (One tailed Mann-U-Whitney test p < 0.008; Fig. 7B). Taken together, we hypothesize that regulatory elements within these two loci are in some way driving their paired association with each other and/or with the PML body.

### Shared regulatory sequences found in loci that frequently associate with PML bodies

**Figure 4:**
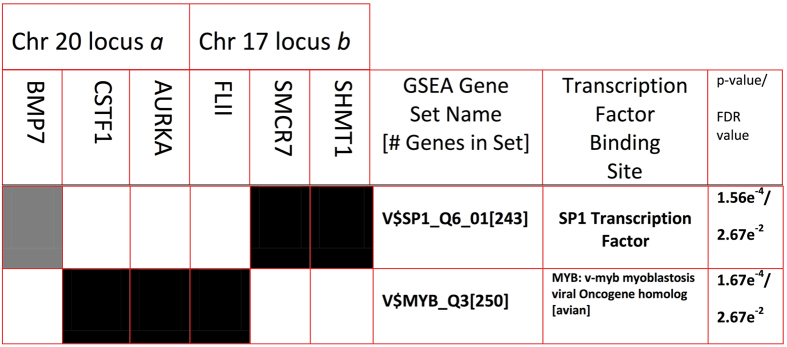
GSEA analysis of Chromosome 20 and Chromosome 17 loci. Significant ontology clustering for genes within 500 kb of chromosome 17 and chromosome 20 hits. Gene sets refer to the GSEA gene annotation^26^ with promoter regions [−2 kb, 2 kb] around transcription start site containing the indicated motif described in the transcription factor column. FDR - False Discovery Rate; Black boxes indicate presence of transcription factor binding site in corresponding row with gene in corresponding column. Grey shaded box indicates presence of degenerate SP1 binding site (GGGCGGR vs. the canonical GGGGCGGGGC) in BMP7.

**Figure 5:**
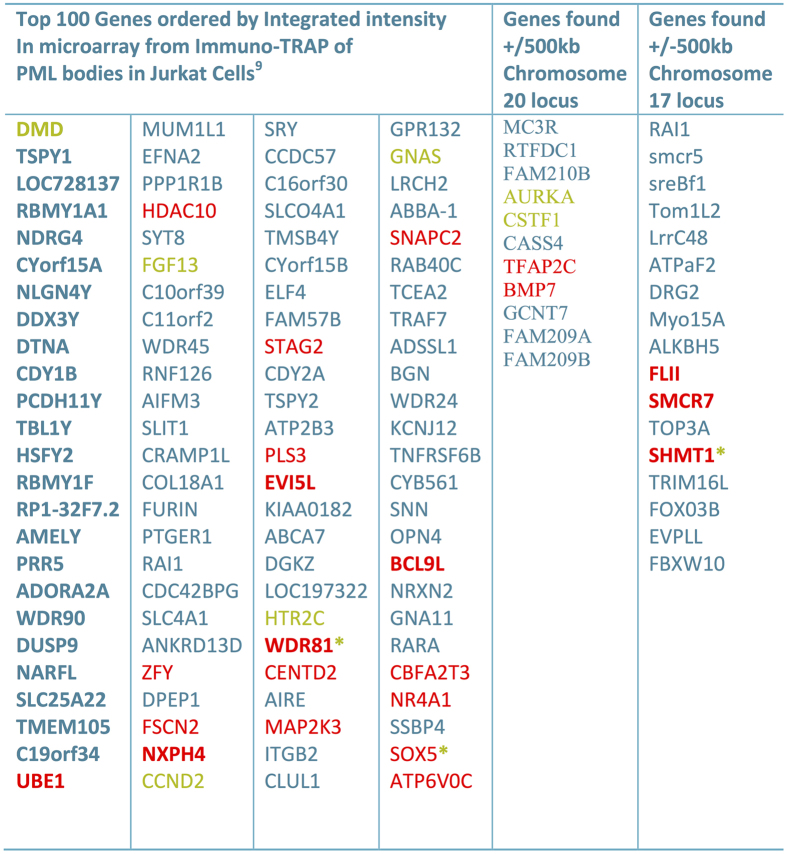
Summary of MYB and SP1 Binding Sites Identified Near Loci Identified by Immuno-TRAP and LTOL. Enrichment of MYB and SP1 binding sites in genes obtained from two different PML body association screens. Genes obtained from both Immuno-TRAP and LTOL were combined and subjected to GSEA analysis. Genes containing MYB consensus sequence binding sites (either NAACNGNCN with 267 sites in the entire genome, FDR < 1.2xe-4; or NNNGNCAGTTN with 250 sites in the entire genome, FDR < 1.07xe-4) in their promoter are highlighted in yellow. Genes with the degenerate SP1 consensus sequence (GGGCGGR; 2940 in entire genome, FDR < 1.18 e-4) are highlighted in red. Genes that also contain the non-degenerate SP1 consensus sequence (GGGGCGGGGC; 243 in entire genome, FDR < 1xe-4), or also contain a MYB consensus sequence, are also in bold and/or asterisked respectively.

As a starting point for this investigation, we explored the possibility that a shared regulatory factor may mediate the interaction with the PML body and with each locus. Using the GSEA ontology database^26^ (see Materials and Methods), we analyzed a 500 kb window on either side of the sequences from chromosome 17 and chromosome 20. Remarkably, there is a significant enrichment of shared consensus sequences for several transcription factors between these two regions. In particular, an enrichment for SP1 and MYB consensus sites were found within the 500 kb window on chromosome 17 (p < 0.0002 and FDR-q value < 0.03; see Figs 4 and 5). Furthermore, the SP1 consensus site is non-randomly distributed throughout the genome, and the cytogenetic band of chromosome 17 which contains the sequence found in our LTOL assay (chr17p11) is the third most highly enriched SP1 consensus site cluster in the genome (p < 0.000175, FDR-q value<0.015). SP1 is a known component of PML bodies that transactivates the targets of the PML/RARα fusion protein in NB4 cells, and its consensus binding site is highly represented in the promoters of the PML body-associated Adenoviral genome^27^,^28^. Moreover, MYB’s interaction with the PML component CBP has been implicated in the differentiation block of promyelocytes^29^,^30^. Hence, we asked whether these consensus sites could be found at other loci that are frequently found associated with PML bodies. We examined the top 100 genes found by probing PML body-associated sequences (obtained by Immuno-Trap)^9^ on a promoter microarray. Indeed, 22 out of 100 genes were significantly enriched in the same SP1 and MYB consensus sites (p value < 0.0000015 and FDR q value <0.00003 SP1 consensus; p value < 0.0000275 and FDR q value <0.00026 MYB consensus; Fig. 5).

**Figure 6:**
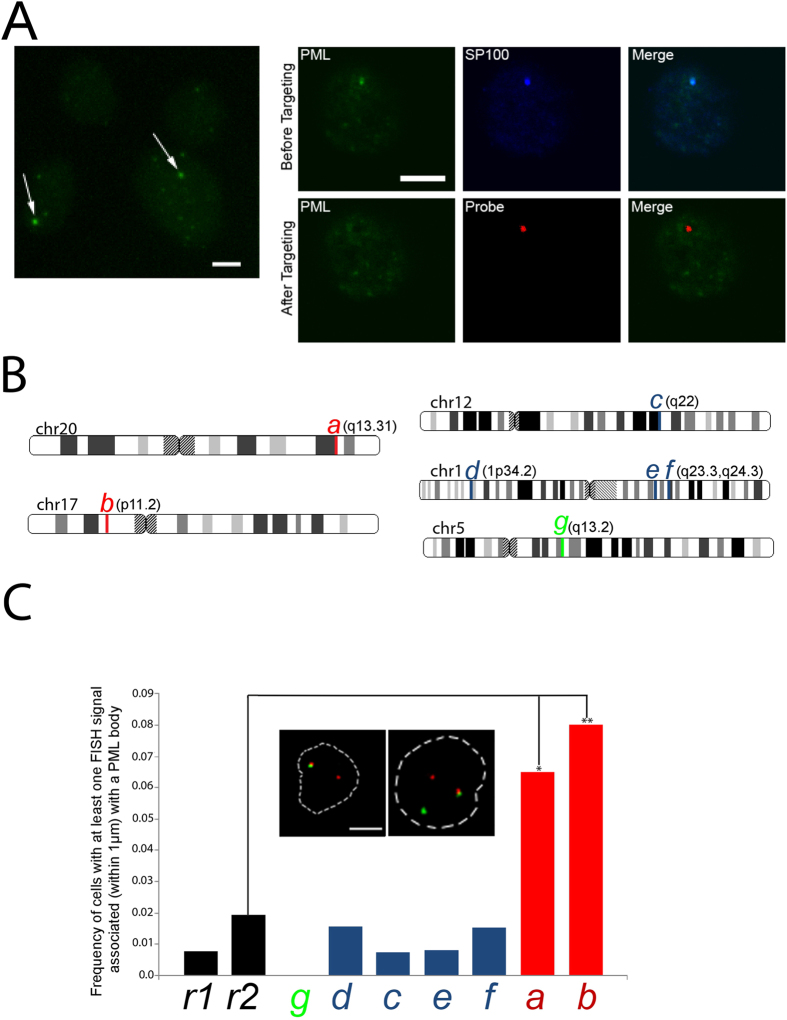
(**A**) Immunofluorescence with PML antibody in NB4 cells. Arrows indicate presence of “aberrant” PML bodies, identified by intense foci in a background of PML-containing structures that do not contain constitutive PML body components. (**B**) Laser targeting of intact PML bodies. i) Immunoflouresence of PML prior to laser targeting. ii) The same cell subsequent to targeted 2-photon irradiation of the PML body. iii) enrichment of fluorescent oligo after subsequent ligation steps. iv) merge of PML and oligo fluorescence to indicate enrichment of oligo at irradiated volume. (**C**) summary of genomic mapping of sequences obtained by laser targeting of single PML bodies. Sequences obtained from the targeting of the same PML body share the same colour in the bar graph and for the vertical lines used to indicate the genomic location of the sequence. (**D**) measurement of 3D distance of FISH signals from PML bodies in NB4 cells indicated a significant association frequency of the hits colocalizing to chromosome 17 and 20 (*p < 0.015; **p < 0.006). Inset: Immuno-FISH of PML (green) association with the chromosome 17 (red; left image) and chromosome 20 loci (red; right image).

**Figure 7:**
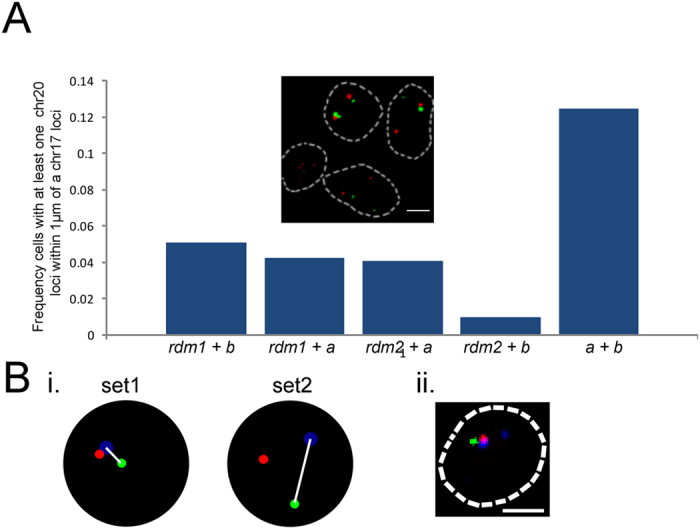
(**A**) Significant frequency of association (i.e. centre-to-centre distance between foci less than 1 μm distance) of chromosome 17 locus with chromosome 20 locus, compared with random controls (*rdm1 and rdm2;* *p < 0.003). Inset: FISH showing interchromosomal association of chromosome 17 (red) and chromosome 20 loci (green). (**B**) i. Mann-Whitney U test was performed to compare the distances of Chromosome 20 locus to a PML body that has a chromosome 17 locus within 1 μm of it (set 1) to those where chromosome 17 is greater than 1 μm to the PML body (set 2). The chromosome 20 locus was significantly closer in set 1 compared with set 2 (p < 0.008). (**B**) ii. Example of the closer association of the chromosome 20 locus (green) to a PML body (blue) where a chromosome 17 locus (red) is within 1 μm of the body.

**Figure 8:**
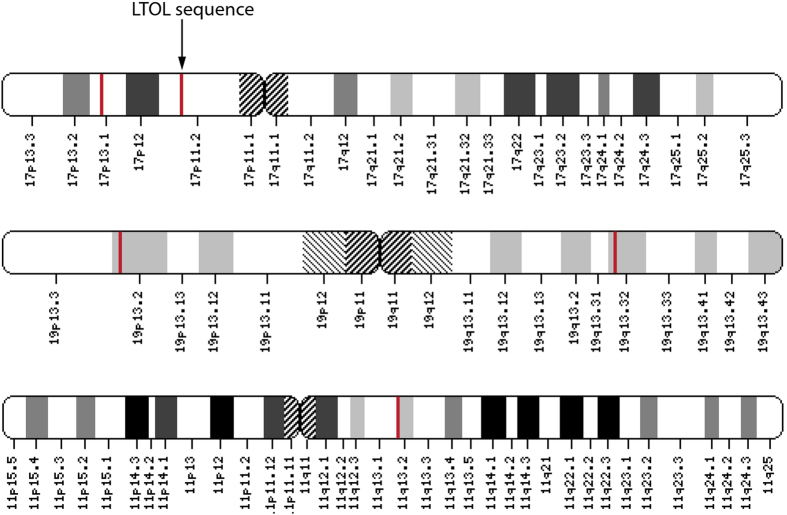
The GSEA database was used to search for chromosomal regions that are enriched in SP1 consensus binding sites. Within significant SP1 cluster sites as annotated by the GSEA database, regions containing at least 3 genes with the SP1 binding site in their promoter within a 100 kb span are indicated by red bars. Note that the chromosome 17 sequence obtained by LTOL in NB4 cells is contained in one of these highly enriched sites.

**Figure 9:**
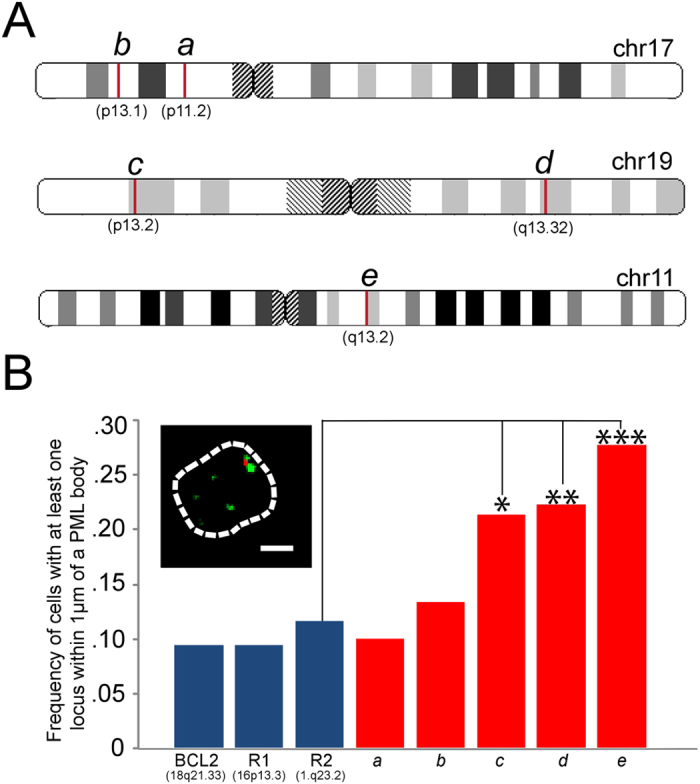
(**a**) Chromosomal locations (indicated by red bars) of SP1 “hotspots”, i.e. containing at least 3 SP1 consensus binding sites within a 100kb span, were identified within the larger regions enriched for SP1 consensus sites as found by GSEA analysis (see Table S2). (**b**) Immuno-FISH was performed in Jurkat cells using probes mapping to chromosomal regions that contain at least 3 genes with SP1 consensus sites in their promoters. A significant association frequency with PML bodies was revealed in 3 out of 5 SP1 “hotspots” compared with BCL2 negative control^9^, and two randomly chosen loci (see Materials and Methods; *p < 0.04; **p < 0.025; ***p < 0.0015). Inset: representative association between a loci (red) and PML (green). (scale bar 5 μm).

As SP1 consensus binding sites were significantly represented in promoters of genes found in previous screen of PML associating genes in Jurkat cells^9^, and as a preliminary investigation into the involvement of the SP1 consensus site in mediating a PML body association, we performed immuno-FISH in Jurkat cells of loci spanning SP1 “hotspots” within SP1 clustered regions, defined as 100 kb spans that contain greater than or equal to three SP1 consensus sites (see Fig. 8). There were five of these genomic hotspots within the GSEA defined SP1 clustered regions. Notably, one overlaps with a sequence obtained from the LTOL targeting of PML bodies in NB4 cells (see Fig. 6 locus “*b*” at chr17p11.2, and Fig. 8). The immuno-FISH revealed three of the five genomic SP1 hotspots significantly associated with PML bodies as compared with negative controls (Fig. 9). Of the two that did not, one overlapped with chromosome 17p11.2, suggesting that, as shown previously in the immunoTrap screen^9^, loci-PML association varies between cell types. Thus, while there are likely additional contributing factors, our initial findings suggest that the presence of clustered SP1 binding sites, although not a sole determinate, may at least contribute to PML localization in a cell-type dependent manner.

## Discussion

Our work demonstrates the feasibility of using targeted 2-photon irradiation to identify gene loci in the vicinity of arbitrary sub-nuclear structures, and that the method is sufficiently sensitive to sequence DNA obtained from targeting a *single* nuclear body from a single cell. We were able to identify sequences within 1 Mb of the expected gene locus at chromosome 6p22.1, shown previously to associate with HLBs^18^. It has previously been suggested that there may be loci other than the histone clusters at chromosome 6p22.1 and 1q21.2 that associate with the HLB^18^. Electron microscopy images show that although the HLB maintains contacts with the surrounding chromatin, it does not appear to contain chromatin at its core, and thus may not be entirely dependent on its association with the histone gene clusters for its formation and maintenance (Supplementary Figure S4). Indeed, our targeting has identified a locus at chromosome 10p15.1 that significantly associates with the HLB as suggested by immuno-FISH in a cell population. It is noteworthy that RBM17, the closest gene to the sequence found at chromosome 10p15.1, codes for SPF45, a known spliceosome component that associates with Cajal bodies, and which is frequently localized adjacent to HLBs^25^,^31^,^32^. As HLBs are also enriched for RNA splicing factors (i.e. NPAT, FLASH), it raises the interesting possibility that this association reflects a novel spliceosome self-regulatory mechanism. Such a regulatory mechanism may be shared by other genes; we have shown that in some cell lines, the *PML* locus associates with PML bodies^9^, and the association of TP53 locus^3^, is notable given PML’s role in modulating p53′s activity^33^. Whether or not there is a functional relationship between the location of the RBM17 gene locus and the gene product’s site of action remains to be tested. In either case, this initial finding underscores the value of the LTOL technique: we have detected a novel, significant association of a gene locus with a nuclear body whose components are either in complex with, or at least functionally related to its protein product.

In its present incarnation, the HLB targeting demonstrates that the accuracy is sufficient to yield sub-chromosomal clusters that frequently associate with a targeted body. Thus, our approach can be used as a starting point for other biochemical approaches to hone in on those specific sequences that may mediate the interaction (i.e. deletion analysis). Improvements to the accuracy and coverage of the targeting procedure can be carried out along several lines. Firstly, the accuracy of this technique is limited by the size of the irradiation volume, and thus may be greatly improved by smaller irradiation volumes afforded by super-resolution microscopy^34^ (Although it should also be realized that nuclear bodies are not rigidly localized, able to jostle or giggle within a defined volume of up to 1 μm in diameter. Hence, at the point of fixation, a gene locus could be completely juxtaposed with a body or separated by the distances indicated by the light microscope)^35^,^36^. Secondly, we may also reduce the residual damage caused by the irradiation procedure (i.e. cyclobutane pyrimidine dimers, strand nicks^37^) that may reduce the efficiency of the PCR amplification. Although it is affected by variations in chromatin packing throughout the nucleus^38^, the frequency of DSBs, given the range of our laser settings (see Materials and Methods), is estimated to occur every 300–900 kb^39^. It has been estimated^40^ that in the range of our laser setting (see Materials and Methods), CPDs are introduced at a frequency of 1 per 1.3 × 10^5^bp and thus are likely interfering with the processivity of the polymerase within a fragment. Treatment prior to PCR amplification with DNA repair enzymes (e.g. Uracil-DNA Glycosylase, and DNA Ligase^41^) could mitigate this residual damage, as has shown to be effective in the amplification of UV damaged DNA obtained in forensic samples (i.e. degraded sample^42^). Thirdly, we also expect that the coverage of sequences obtained from the targeting is limited by dominant amplicons that emerge during early cycles of the PCR. A modified approach based on a limited number of rounds of linear amplification followed by deep sequencing would likely improve our coverage. Even so, the amount of *bona fide* sequences obtained per experiment is sufficient to detect specific loci-NB associations, and furthermore, we were able to obtain sequences from multiple chromosomes from the targeting of a *single* nuclear body.

As is uniquely afforded by the LTOL approach, through determining DNA sequences in the vicinity of a *single* nuclear body, we have found convergent loci that share commonality among the transcription factor binding sites significantly enriched in them. A similar finding of gene convergence at subsets of transcription factories that are enriched in their cognate transcription factors^43^, leads us to conclude that the spatial association of co-regulated genes at a shared nuclear body is a common theme in nuclear organization, and may underlie a novel co-regulatory mechanism. If so, we propose LTOL as a crucial method that can be generally applied towards detecting this phenomenon at other unique nuclear body subtypes.

We propose that this laser targeting technique will also have wide applicability as a complement to ChIP and immunoTRAP, particularly for those questions of chromatin association with nuclear substructures that preclude a population-based or biochemical pulldown approach. For example, the nuclear body in question may be insoluble, the chromatin contacts transient or not mediated by the precipitated protein, or the constituent protein is present at nuclear sites other than the nuclear body. Furthermore, as is underscored by a recent report of cell-to-cell variation in large-scale chromatin structure^44^, single cell approaches are crucial to revealing those rare or transient cellular events that would otherwise be undetected in a population-based assay. The “unique” one or two intact PML bodies in NB4 cells present such a challenge to conventional biochemical approaches, since their constituent proteins are found in other PML-containing micro-foci in the same nucleus. They are also particularly interesting candidates as “gene hubs”, because their integrity may be maintained by their affinity for specific chromatin contacts^14^. Our initial targeting has so far revealed two loci that both frequently associate with PML bodies, and with each other. The specific sequences at these loci that are responsible for these associations are not known. However, our finding of shared transcription factor binding consensus sites between these loci lead us to hypothesize that shared regulatory factor(s) may mediate the interaction with the PML body and with each locus. Given its role in the differentiation of promyelocytes^29^,^30^, we are particularly interested in the shared enrichment between the two loci for the MYB transcription factor consensus sequence (see Fig. 5). The enrichment of SP1 consensus sequences at the chromosome 17 locus, and its significant enrichment in loci identified at PML bodies by immuno-Trap^9^, demonstrates the requirement for further exploration in light of its known role as a transactivator through PML-RARα in NB4 cells^27^.

Our work here serves as a proof-of-principle for the laser targeting technique, demonstrating its advantages for interrogating the chromatin neighbourhood of single arbitrary sub-nuclear structures. Although we were motivated to develop it for our own questions of PML biology, because of its power in generating and testing hypotheses without *a priori* knowledge of a candidate locus, we recommend LTOL as a critical method to apply to similar questions of gene locus association with other sub-nuclear structures and domains. We expect that it will find wider use for the detection of chromatin/nuclear body interactions that so far are intractable with present methods, as it does not require specialized equipment beyond that found in most institutional microscopy facilities. We have also recently developed a parallel approach using nano-dissection tools inside a scanning electron microscope^45^. Together, these two new methods serve as the first demonstrations of DNA isolation from *single* nuclear substructures *in situ*.

## Methods

### Preparation of PEN coverslips

PEN coverslips were prepared as follows: A small “keystone” shape was etched into 25 mm round glass coverslips using a diamond knife and overlayed stencil. The keystoned coverslips were washed and dried in 90% alcohol. Sheets of 1.35 μm PEN film (kindly provided by PALM) were cut in approximately 18 mm square pieces and floated on nuclease free water. The keystone coverslips were brought underneath the floating PEN film and lifted out in order that the PEN film is evenly overlaid on the coverslips with the etched keystone roughly in the centre of the PEN film. The “PEN coverslips” were then dried in a 60 degree oven overnight. Rubber cement was then applied to the border of the overlaid PEN film and dried at room temperature overnight. Prior to cell culture the PEN coverslips were UV irradiated in a biohood for 30 minutes.

### Immunolabelling/Preparation for Laser Targeting

Cells were incubated on PEN coverslips overnight. After a rinsing out media with phosphate buffered saline (PBS), Cells were fixed for 10 minutes with 4% paraformaldehyde (PFA) in PBS. Cells were then washed three times for five minutes each in PBS, and permeabilized for five minutes with 0.1% Triton X-100 in PBS followed by three more PBS washes of five minutes each. In order to prevent endogenous DSBs from being substrates for probe ligation and subsequent amplification, we blocked cells with a 13bp hairpin oligo (5′ GCG CTA GAC C*G GTC TAG CGC 3′; *internal Cy5 conjugate) that did not contain sequences that are complementary to the primers used for the amplification procedure. We followed the “*Oligo Ligation of Targeted Double Strand Breaks”* protocol as detailed below except that the hairpin blocking oligo was used in place of the targeting probe see “*Details of Oligos*”. Cells were then immuno-labeled as previously described^27^ using either mouse anti-NPAT (Abcam; HT1080 cells) or rabbit anti-PML (PGM-3, Santa-Cruz; NB4 cells) primary antibody and anti-Rabbit (NB4 cells) or anti-mouse (HT1080 cells) alexa488 secondary antibody (Invitrogen). Cells were then incubated with Hoechst 32258 diluted to 0.5 μg/mL in PBS, and covered in tin foil to avoid light exposure.

### Induction of DNA Damage in Subnuclear Regions

Using a confocal fluorescence microscope (LSM510 META; Carl Zeiss MicroImaging, Inc.) equipped with an argon laser tunable to 458, 488, and 514 nm transmission, and a Chameleon two-photon laser transmitting a maximum power of 1300 mW at 780 nm (Coherent), keystones were located and situated under the objective either by direct visual inspection or under white light through a 10X Plan-Neofluoar NA 0.3 objective. The field was scanned with 488 nm excitation, visualized through a short pass filter (505–530 nm) with the 10X objective, and a low magnification image was taken. A chosen cell or cluster of cells within the keystoned was visualized as above under the 63X C-Apochromat NA 1.4 objective, and a high magnification image was taken. Using the LSM 510 photobleaching software, regions of interest (ROIs) were drawn in the high magnification image corresponding to subnuclear regions of the chosen cell(s) to dictate the path of the two-photon laser. The ROIs were irradiated by femtosecond 780 nm pulses from the Chameleon laser, for an effective two-photon absorption event of 390 nm. Various laser settings (combinations of 25%, 30%, 40% transmission power with 25, or 50 iterations per pixel) were used in the initial labeling experiments. In the targeted laser damage and subsequence amplification of DNA extracted here, a laser power of 30% with 50 iterations per pixel was used. Single nuclear bodies were targeted as follows: a pinhole size corresponding to an optical section of 0.7 μm was used to take an image under 488 nm excitation. Using ImageJ, an 8-bit grey scale 512 × 512 image was created from a mask of the original image after thresholding to the minimum level of the chosen chromocentre’s fluorescent signal. The pixel corresponding to the centre of mass of the chosen chromocentre’s fluorescence signal in the masked image was located using an automated macro, and the location of this pixel was encoded as a binary 512 × 512 array or “text image”. A Zeiss macro then positioned the laser pulse according to the resultant text image, and a 750 nm two-photon pulse was applied for a duration of 1 second at approximately 900 mW with AOM attenuation set at 4%. A subsequent image was taken under 488 nm excitation to confirm that the irradiated spot was accurately targeted (the 750 nm two-photon irradiation is sufficient to bleach the alexa 488 signal in the targeted region).

### Oligo Ligation of Targeted Double Strand Breaks

To ligate the oligo (either hairpin or double stranded) to the laser targeted double strand breaks, the protocol of Didenko *et al*.^20^ was followed with slight modifications. For the sake of illustration, any original details of the protocol that have been changed since its optimization are indicated in square brackets ([]). After the laser damage, the cells were washed three times in PBS for five minutes each, and incubated for 1 hour [30 minutes] at 37 °C in Klenow buffer (70 mM Tris HCl pH 7.5, 70 mM MgCl2, 1 M dithioerythritol (DTT)) with 100 U/mL, and 2.5 mM each of dGTP, dATP, dCTP, and dTTP. After the Klenow reaction, the cells were washed three times for five minutes each in PBS. Next, the cells were incubated for ten minutes in ISOL buffer (1X T4 DNA ligase reaction buffer (Fermentas) supplemented with 15% polyethylene glycol 8000 (PEG 8000), 0.5 mM adenosine triphosphate (ATP), and 0.05 mg/mL bovine serum albumin (BSA)). The cells were then incubated with ISOL buffer with the addition of 100 U/mL T4 DNA Ligase (Fermentas), and the appropriate oligo (hairpin oligo 35 μg/mL, double stranded oligo 0.29 nM). After 18 hours [3 hours], were then washed 3 × 5 minutes with 0.1% Tween 20/2XSSC solution at 42 °C, and 3 × 5 minutes at 60 °C with 0.5XSSC solution. The efficiency of the ligation is confirmed by the Cy3 signal under fluorescence microscopy, and then prepared in preparation for the microdissection as follows: the cells were dipped for two minutes in a filter purified 5% dilution of hematoxylin (Sigma) in PBS, washed in nuclease free water for 30 seconds, dehydrated for two minutes each in 70%, 90%, and 100% ethanol dilutions, and then air dried for one hour.

### Details of Oligos

The sequence of the hairpin is: 5′ GCG CTA GAC C*G GTC TAG CGC 3′, where the asterisk denotes an internal Cy5 conjugate. The Cy3 conjugated double-stranded targeting oligo used for the ligation of DSBs subsequent to induced laser damage is: Strand 1- 5′ (Cy3) AGT GGG ATT CTT GCT GTC AGT TA**G CTG** 3′, strand 2- 5′ CAG CTA ACT GAC AG(ddC) 3′ (ddC: dideoxy C). The nucleotides in bold indicate the “signature sequence” used to indicate a bona-fide ligation event (Fig. 1 and Supplementary Figure S1) Note that this oligo Linker contains the priming sites for the PCR amplification. (adapted from Langer *et al*.)^46^.

### Microdissection of Cells and DNA Extraction

After probe ligation, targeted cells were visualized using the Zeiss LSM 510 microscope to confirm enrichment of probe ligation in the targeted region. A low magnification (10X objective) bright field image of the keystone was taken and annotated with the location of the targeted cells. In preparation for the microdissection, the cells were stained with hematoxylin as follows: cells were dried at 37 °C for thirty minutes, dipped for two minutes in a filter purified 5% dilution of hematoxylin (Sigma) in PBS, washed in nuclease free water for 30 seconds, and then air dried for one hour. For microdissection, a PALM (PALM Microsystems inc.) LMPC microscope was used. The targeted cells corresponding to the annotated low magnification bright field image were located in the keystone under the 40X objective of the microscope. The targeted cells were catapulted into the cap of a 200 μL nuclease - free eppendorf tube (Ambion) containing 4.5 μL of DNA extraction buffer: 0.5 μl of 10 One-Phor-All-Buffer- Plus (Amersham Pharmacia Biotech), 0.13 μl 10% Tween 20 (Sigma, Germany), 0.13 μl 10% Igepal CA-630 (Sigma), 0.13 μl Proteinase K (10 mg/ml, Sigma). The eppendorf tube with lysis buffer and suspended cell in cap was then incubated in a PCR machine with a heated lid at 42 °C and block temperature at 70 °C for 16 hours. The reaction was then spun down and heated to 80 °C for 10 minutes in order to inactivate the Proteinase K.

### DNA Extraction and PCR Amplification

We adapted the protocol of Langer *et al*.^45^ as follows: PCR is performed in 50 μl total volume using the Titanium Taq polymerase kit (Clonetech) under the following conditions: 72 °C 1 minute, 68 °C 3 minutes, then 14 cycles of 94 °C 40 seconds, 57 °C 30 seconds, 68 °C 1 minute 30 seconds increasing by one second each cycle. Then 8 cycles of 94 °C 40 seconds, 57 °C 30 seconds, 68 °C 1 minute 45 seconds increasing by one second each cycle. Then 22 cycles of 94 °C 40 seconds, 65 °C 30 seconds, 68 °C 1 minute 53 seconds increasing by one second each cycle. Then 68 °C for 3 minutes 40 seconds. Primer sequence is as follows: 5′ AGTGGGATTCTTGCTGTCAGTTA 3′. Then 10 μl of the amplification product is analyzed on a 2% agarose gel, and 2 μl is cloned for sequencing using the CloneJET PCR cloning kit (Thermo Scientific).

### 3-D FISH and Imaging

FISH was performed as previously described^9^. BACs corresponding with the mapped location of HT1080 sequences (Fig. 2B) and NB4 sequences (Fig. 3B) are detailed in Supplementary Table S1. Random BACs were each obtained using two rounds of a random number generator that yielded a particular chromosome coordinate: the first random number generated with integer values restricted between 1 and 23 chose the chromosome, and the second random number was restricted to integer values between 1 and the bp length of the chromosome chosen in the first round. Confocal stacks with a Z increment of 0.1 μm were taken with an Olympus IX81 microscope.

### 3-D FISH Measurements and Statistics

The percentage of cells with at least one BAC signal associating with a PML or HLB NB (using distance thresholds as indicated) was determined by measuring the Euclidian center to center distances between FISH signals and PML with ImageJ. Significant differences (p < 0.05) in association frequencies were determined by taking the two-tailed p value under an exact Fisher test. To determine if there was a relationship between the association of one locus at a PML body with the association of the other locus with that same body, we compared the distances of locus *a* (or *b*) to a PML body that had locus *b* (or *a*) within 1 μm of it, with the distances of locus *b* (or *a*) to a body that did not have a locus *a* (or *b*) within 1 μm of it. A Mann-Whitney U test was performed on these two sets of distances. To calculate lower estimate for the p value associated with finding two sequences within 2 MB of the two histone cluster on chromosome 6 (P_insidewindow_), we considered it as the complement probability of a sequence *not* being within a 4 Mb window centered on the histone cluster (P_outsidewindow_). This gives us: P_insidewindow_ = 1−P_outsidewindow_ = 1−[((number of base pairs in human haploid genome) − (4 Mb))/(number of base pairs in human haploid genome)]  ≈ 0.0013. We then used this value as the probability of success in a binomial test. To determine a p value for the enrichment of chromosome 6 sequences obtained from the HLB dissections, we considered an upper estimate of the chance of getting a sequence from chromosome 6 as: (size in base pairs of chromosome 6)/(size of haploid genome) ≈ (171 × 10^6^)/(3 × 10^9^) = 5.7 × 10^−2^. We then used this value as the probability of success in a binomial test.

### GSEA Analysis

A 500 kb window on either side of the loci corresponding to the sequences obtained from LTOL that map to p11.2 and q13.31 on chr17 and 20 respectively (Fig. 6D) were entered into the GSEA database to search for the presence of consensus binding sites for transcription factors. An FDR-q threshold value of 0.05 was used to determine enrichment; this takes into account the probability of a binding site occurring within the queried region as a function of its relative frequency throughout the genome, as well as the probability of *any* transcription factor occurring within the queried region^47^.

## Additional Information

**How to cite this article**: Anchel, D. *et al*. A novel single cell method to identify the genetic composition at a single nuclear body. *Sci. Rep.* **6**, 29191; doi: 10.1038/srep29191 (2016).

## Supplementary Material

Supplementary Information
