# Supplementary material for: A novel single cell method to identify the genetic composition at a single nuclear body
Source: Sci Rep. 2016 Jul 8;6:29191. doi: 10.1038/srep29191 (PMC4937434; doi:10.1038/srep29191)
Supplement: Supplementary Information [file srep29191-s1.pdf]

A novel single cell method to identify the genetic composition at a single nuclear body

David Anchel, Reagan W Ching, Rachel Cotton, Ren Li, David P Bazett-Jones*

Supplementary Figure S1. a) Gel result after laser targeting and amplification. Shown are combined amplicons from three targeting experiments of HLBs. Two lower and upper left most lanes: 1kb, 300bp ladders; lanes 1-5: Negative controls (lysis buffer); lanes 6-10: Non targeted cells from the same coverslip as targeted cells; lanes 11-23: each lane corresponds to a single targeted and microdissected cell that was targeted at a signal HLB. b)i. genomic sequence (green) that originated from a bona-fide ligation event is distinguished by a primer sequence (blue) and nesting "signature" sequence (pink). Successful amplicons are counted for those that occur either by primer recognition for its complimentary sequence on two ligated probes flanking a genomic span, or by mispriming on one end. ii. Occasionally amplicons do arise both in lysis buffer controls (as seen in lane 1), and non-targeted cells however these were discounted by a lack of signature sequence. c) Targeting in cells away from an HLB. i. An HLB body indicated by green foci prior to two-photon irradiation (see Materials and Methods). ii. After irradiation away from the HLB body, the HLB body remains unbleached (compare green foci between i. and ii.). iii. The same cell after ligating an oligo (see Materials and Methods), an enriched focus of fluorescent oligo (red) can be seen that does not colocalize with an HLB body. iv. Merge of the red oligo channel and green HLB channel confirming that the targeted region does not colocalize with an HLB body. Although no *bona fide* sequences were obtained from this additional control experiment (i.e. those that contained a signature sequence), the enrichment of sequences that cluster to

the histone locus was only seen upon the targeting at HLB bodies, indicating that it is not an artifact of the targeting procedure itself.

Supplementary Figure S2. Because the effective wavelength necessary to induce two-photon breaks (390nm) is confined axially to the focus of where the 750nm irradiation in which the 2-photon interactions occur (see Material and Methods). Shown here is a z-stack of a targeted cell merging the probe signal (red) with the H3K9me3 signal (green). The enriched probe signal is largely confined to between the 1.4μm and 2.24μm frames. (scale bar 1μm).

Supplementary Figure S3. Boxplots of center-to-center distances in microns, comparing those loci that show a significant frequency of association (as illustrated in Figures 3 and 4) vs. random control loci to HLBs (a) and NB4 bodies (b) respectively. Represented by the box are the second and third quartiles, with the whiskers representing the minimum and maximum values, the middle line representing the median, and "X" the mean. The italicized names of the loci correspond to the respective italicized names in Figures 3 and 4.

Supplementary Figure S4. ESI (electrospectroscopic imaging[47]) of HLB body. (a) HLB body is seen as protein rich (blue) structure making contacts with surrounding chromatin (yellow), but devoid of chromatin itself. (b) Phosphorous channel alone reveals puncta

within the HLB structure (arrows) suggesting the presence of RNA. Scale bars 200nm.

Supplementary Figure S5. Relative Radial Distances of chromosome 17 and 20 loci measured from the center of nuclei of NB4 cells. Distances of FISH signals using BACs corresponding to 17 and 20 loci (see Table S2) were measured to the center of the nucleus, and normalized by nuclear volume. Radial distances of PML signals to the center of the nucleus were similarly measured. Plotted are mean radial distances, error bars represent standard deviation.

Supplemental S1

A

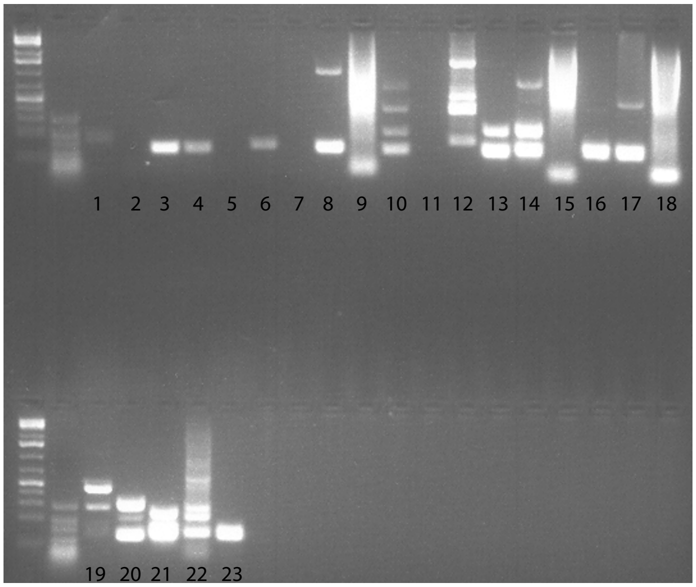

B

i. Chromosome 6 28658264-28658322

GGGATTCTTGCTGTCNGTTAGCTGACGTACCTACTTTC
NGTACNNNNNNNNNANNNACTTATCACATGGTAAGTG
GCTGTCAGTTAGCTNNNTNNNNNANCTGCAGCTAAC
TGACAGCAAGAATCCC

ii. Mispriming

GGGNNTCTTGCTGTCAGTTAGGGACTAAAGAAAATGT
NNNNCNNANTTTGATTTGCTTGCTGATCTTCGGTTAA
CTCCATNNANNANNTNTGTACTGATTCAGTTAACTGA
CAGCAAGAATCCC

C

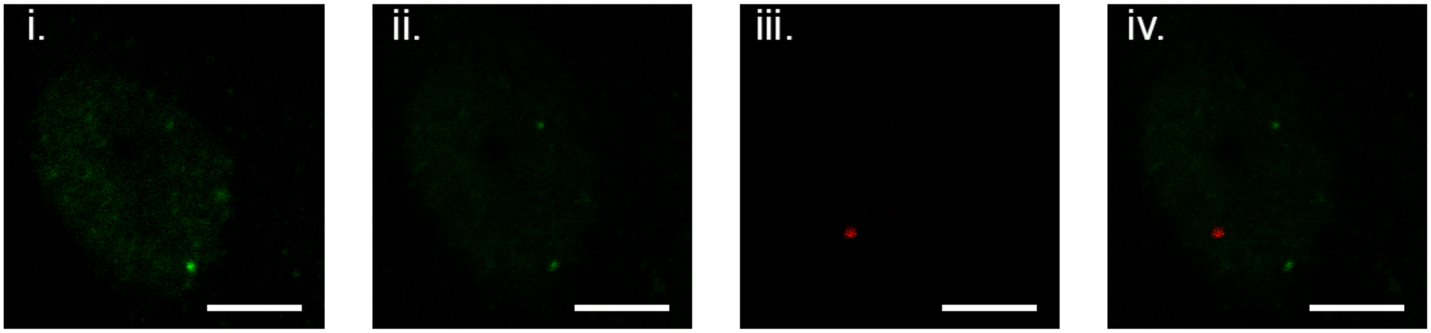

Supplemental S2

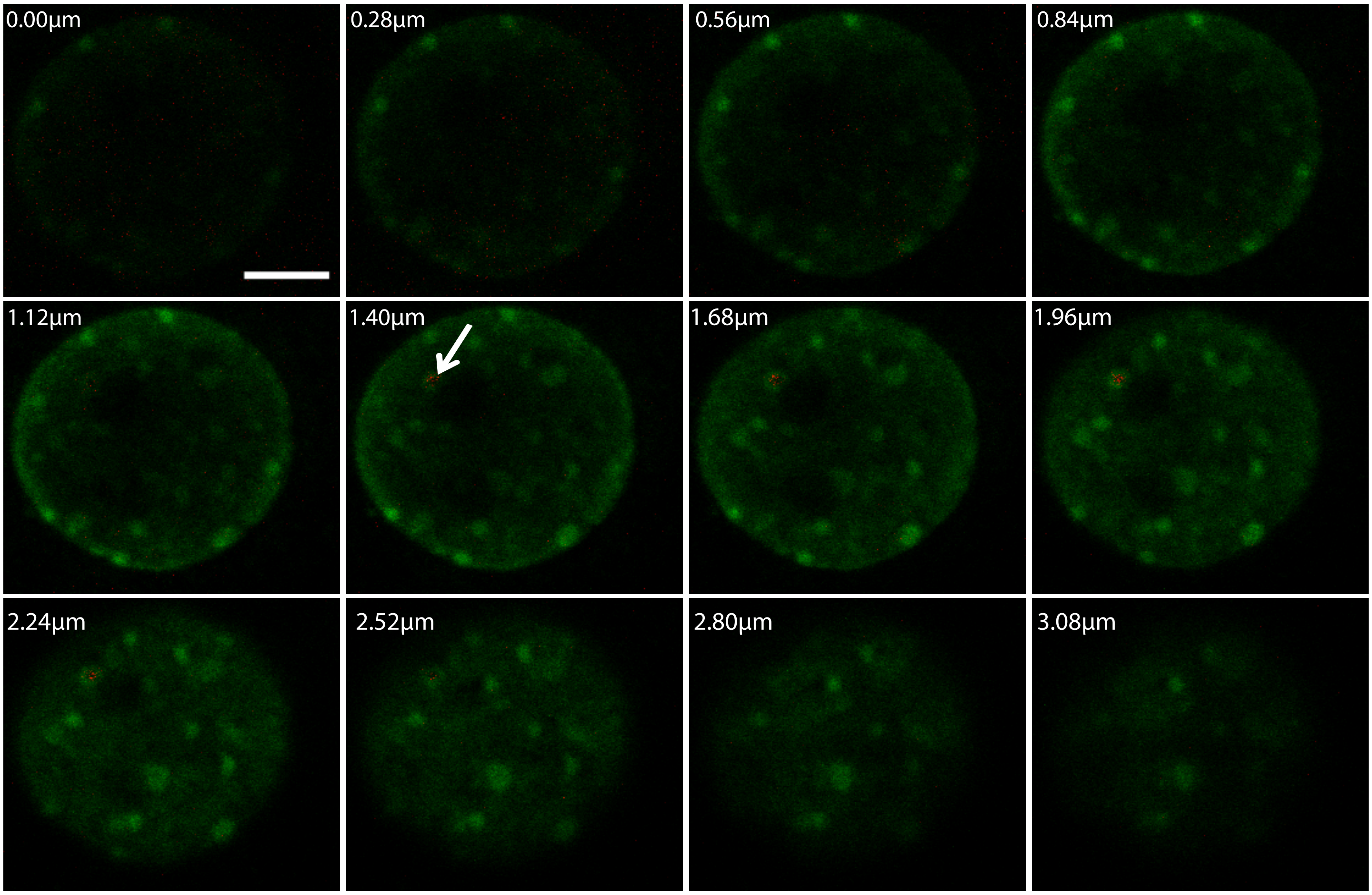

Supplemental S3

A

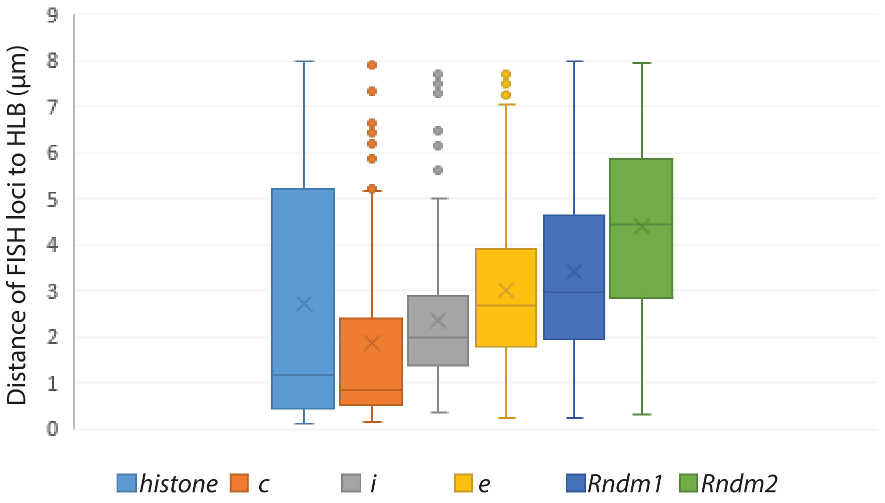

B

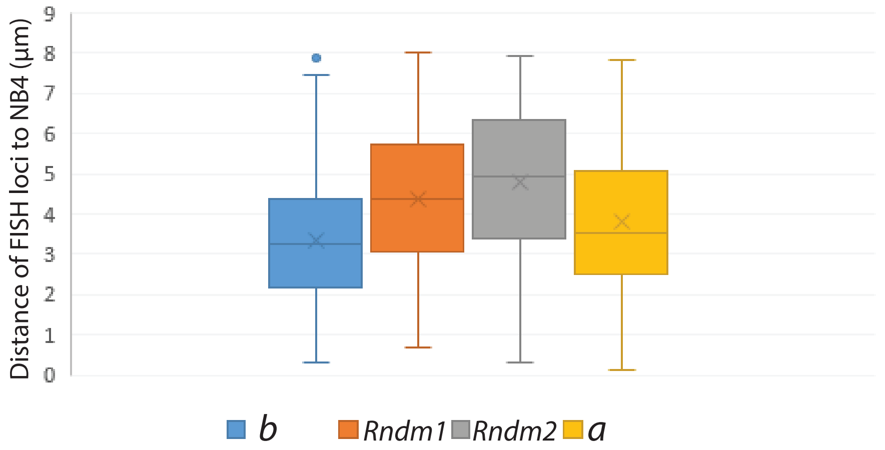

Supplemental S4

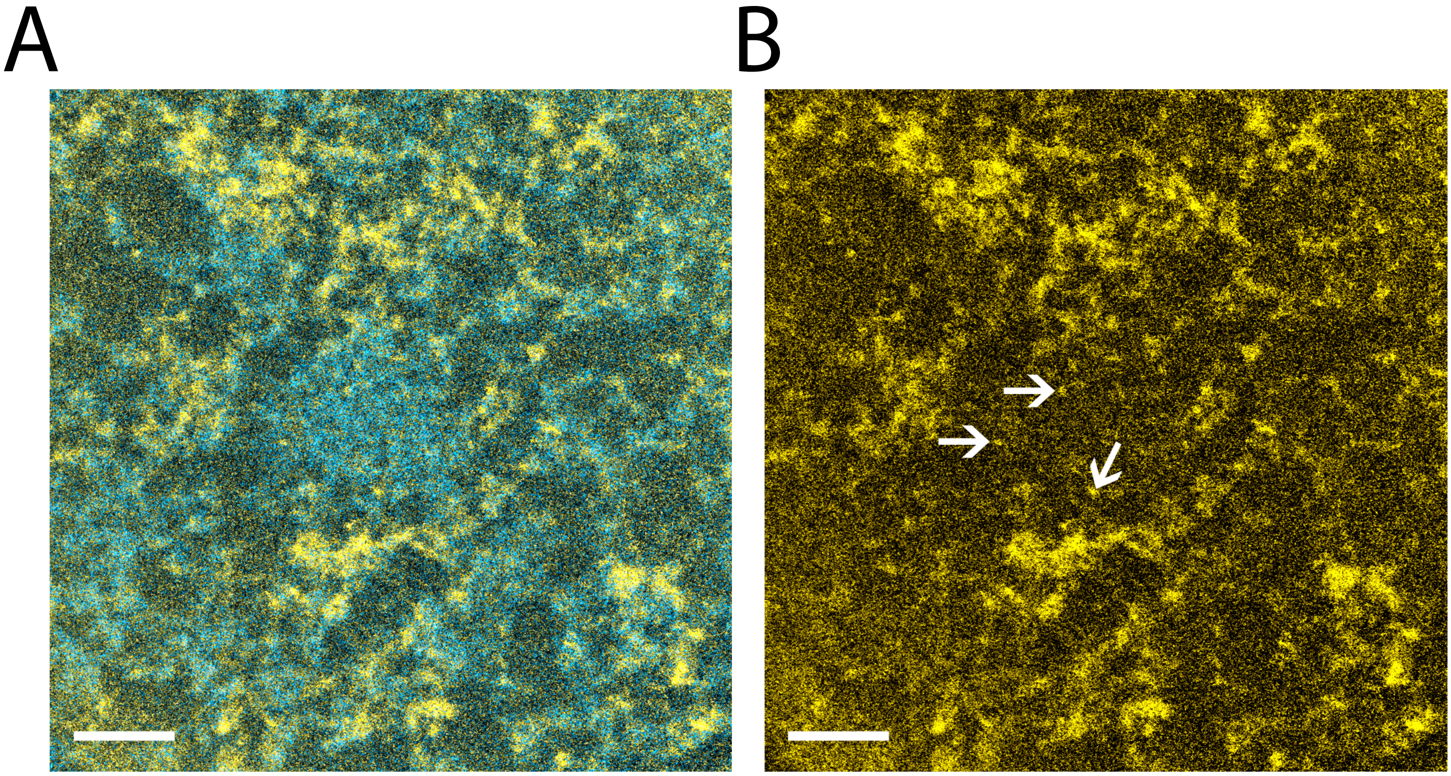

Supplemental S5

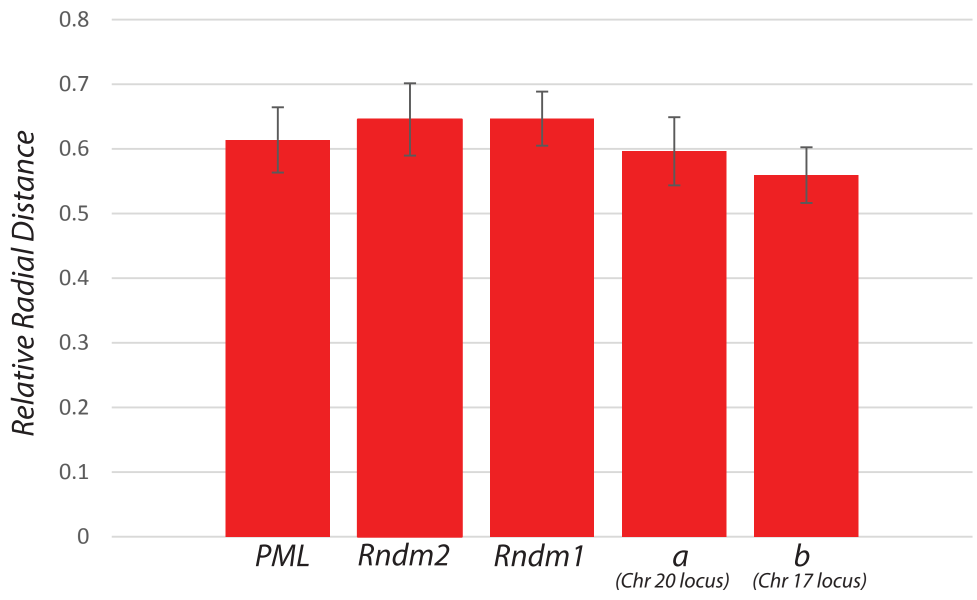

Table S1. List of BACs used in this study

Map designation (from Figure)	Overlapping BAC	Number of cells analyzed by FISH
a(2,3)	Rp11-642E22	118
b(2,3)	Rp11-24C3	68
c(2,3)	Rp11-1145O11	97
d(2,3)	Rp11-12N18	99
e(2,3)	Rp11-609G19	83
f(2,3)	Rp11-430O23	65
g(2,3)	Rp11-1147O22	64
h(2,3)	Rp11-836G11	92
i(2,3)	Rp11-14P15	93
histone(3)	Rp11-2P4	98
Rdm1(3)	Rp11-314G12 (14q22.3)	85
Rdm2(3)	Rp11-1149E8 (12p13.31)	104
a(4,5)	Rp11-145H11	154
b(4,5)	Rp11-936C14	137
c(4)	Rp11-100C13	133
d(4)	Rp11-164P9	127
e(4)	Rp11-254M15	125
f(4)	Rp11-768D16	130
g(4)	Rp11-493D24	76
Rdm1(4,5)	Rp11-79E8 (8q24.21)	132
Rdm2(4,5)	Rp11-89D23 (13q32.1)	103
a(7)	Rp11-936C14	159
b(7)	Rp11-1D5	120
c(7)	Rp11-145L1	112
d(7)	Rp11-91A12	130
e(7)	Rp11-856B14	119
Rdm1(7)	Rp11-192K1	138
Rdm2(7)	Rp11-574F21	138
BCL2(7)	Rp11-299P2	96
Rdm1(S5)	Rp11-79E8 (8q24.21)	30
Rdm2(S5)	Rp11-89D23 (13q32.1)	30
a(S5)	Rp11-145H11	31
b(S5)	Rp11-936C14	31

Table S2. List of genes enriched for Sp1-binding sites found but GSEA

MyB containing genes	SP1 containing genes	d-1p34.2 chr1:38186980-38686980	g-5p13.2 chr5: 37265765-38265765	e-254M15 chr1:162132340-162632340	f- 768D16 chr1:182833429-183333429	c-100C13 chr12: 99028773-100028773
ABHD15	ABCC1	INPP5B	WDR70	ATF6	RNASEL	ANKS1B
ABHD2	ACBD5	SF3A3	GDNF	OLFML2B	RGS16	FAM71C
ACCN1	ACE	FHL3	NUP155	OLFML2B	LOC284648	LOC101928937
ACTC1	ACER3	UTP11L	EGFLAM	NOS1AP	RGS8	
AGER	ACSF2	POU3F1		NOS1AP	NPL	
AHNAK	ADAM15	MIR3659		MIR4654	DHX9	
ALDOA	ADAM17	LINC01343		MIR556	SHCBP1L	
ANKRD17	ADCK1	RRAGC		NOS1AP	LAMC1	
ANKRD28	ADSS	GJA9-MYCBP		C1orf226	LAMC2	
ANKZF1	AGBL5	MYCBP		C1orf226	NMNAT2	
ARHGEF6	ANKRD12	GJA9		SH2D1B	SMG7-AS1	
ASB7	ANP32A	RHBDL2		UHMK1	SMG7	
AURKA	APLP1			UHMK1	NCF2	
B3GALT2	APLP2			UHMK1		
BAP1	ASCL2			UAP1		
BDNF	ASPHD1			DDR2		
BEND4	ATP2A2			HSD17B7		
BHLHE22	BCL2L2			CCDC190		
BSCL2	BCL7C					
C12orf65	BCL9L					
C14orf43	BRI3BP					
C17orf102	BTBD10					
C19orf47	C11orf30					
C1orf21	C11orf35					
C4A	C11orf68					
C4B	C12orf66					
C6orf138	C17orf28					
C7orf62	C17orf57					
C9orf93	C17orf61					
CACNA1G	C1orf122					
CACNB3	C1orf43					
CADM1	C2CD2L					
CADM2	CACNA1A					
CAMK2A	CADM1					
CANX	CALM3					
CASK	CAMK2G					
CCL5	CBLN1					
CD68	CCDC85B					
CDCA3	CD3EAP					
CDH20	CDC37					
CDK2	CDK5R1					
CEBPB	CHAT					
CELF1	CHKA					
CHGA	CLPTM1					
CNOT2	CNNM4					
CNTF	CORO1C					
COL12A1	CPD					
COX7A2P2	CPSF7					
CRISPLD1	CRY1					
CSDE1	CSNK1D					
CSTF1	CSPG4					
DDIT3	CSRP3					
DGKI	CTTNBP2NL					
DKFZp761E198	CXCL12					
DMD	CYP26B1					
DNAH12	DAK					
DSCAM	DCAF4					
E4F1	DDB1					
EHMT1	DET1					
EPB41	DGKA					
ERGIC1	DKFZp761E198					
ERRFI1	DNAJC4					
ESRRA	DRAP1					
ESRRG	DUOX1					
ETV4	ECE1					
FAM13B	EEF1DP3					

FAM63A	EFNA3						
FGF12	EFNB3						
FIGN	ELAC2						
FLI1	ELAVL3						
FOXF2	ELL						
FOXP1	EML3						
FRAS1	EPS15						
FXR2	EPS8L2						
GAN	ERC1						
GBA2	ERO1LB						
GCAT	EVI5L						
GMFG	FAM100B						
GMPR2	FAM160A2						
GNAS	FDX1						
GNAT1	FGF11						
GNG3	FKBP8						
GNL1	FLII						
GOLGA7	FOSB						
GPHN	FRMD5						
GRK5	GABARAPL2						
GTF3C2	GAS7						
HCN1	GGN						
HEATR7B2	GLTP						
HEBP1	GOLGA3						
HERPUD2	GPR3						
HHEX	GRB2						
HMGA2	HCN4						
HNRNPF	HES7						
HOXA11	HOXC13						
HOXB3	HSPB9						
HRH4	HTR7						
HSPA9	IDH3A						
ID4	IGF2BP1						
IER5L	INPPL1						
IL11RA	IQGAP1						
ING3	IRX3						
KLF12	JUB						
KLF5	KANK2						
KRT25	KAT2A						
LAMA3	KAT5						
LEP	KCNQ4						
LHX6	KIAA0528						
LIG4	KLF11						
LINS	KLF2						
LMNA	KLF5						
LNPEP	KTN1						
LOC148872	LASP1						
LTBP1	LDB1						
LUC7L3	LIMA1						
LYN	LLGL2						
MAP4	LOXL4						
MAZ	LPPR2						
MBD6	LRRC8E						
MDGA2	LYRM1						
MED13	LZTS2						
MGLL	MAP2K7						
MIR22HG	MAP3K11						
MLL2	MAP3K6						
MLL5	MAP4K2						
MLN	MAPK7						
MXD3	MAZ						
MYL6B	ME3						
MYO18B	MIR22HG						
MYOZ2	MYO19						
NCOA5	MYO1C						
NDEL1	NAV1						
NDUFC1	NET1						
NEDD8	NFAT5						

NEUROD6	NFYB					
NIPBL	NLK					
NMUR1	NOTCH3					
NOVA1	NPAS4					
NR3C2	NTF4					
NRF1	NUFIP2					
NRGN	NXPH4					
NRXN3	OAZ2					
OAZ2	OTX1					
ODF2	PAK4					
OMG	PARD6A					
OPCML	PCGF2					
ORAI3	PCYT2					
ORMDL2	PDIK1L					
P2RY2	PER1					
PAN2	PEX14					
PCDH9	PGF					
PCSK1N	PGM2L1					
PDAP1	PHF23					
PHF7	PHOX2A					
PIK3CG	PIAS1					
PIK3R3	PIAS3					
PLXNC1	PIGN					
PMEL	PIGV					
PNMA1	PIGW					
PNOC	PIP4K2B					
PPARGC1B	PITPNA					
PPP4R4	PITPNM1					
PRDM13	PLEKHM1					
PRDM16	POLR2I					
PREB	POLR3E					
PRICKLE1	PPM1J					
PRPF38B	PRMT1					
PRR3	PSMC6					
PRUNE	PTCH2					
PTMS	PTOV1					
RAB2A	PTPN2					
RAPSN	PUSL1					
RARG	PVRL2					
RASA3	RAB26					
RBM39	RAB2B					
RDH11	RAB35					
RFX3	RABEP2					
RGS2	RBFOX1					
RHOBTB3	RCOR2					
RIMS1	RELA					
RIN1	RELB					
RPL4	RHBDL3					
RREB1	RHOG					
RUNX1T1	RND2					
SCN3B	ROM1					
SEC16B	RTF1					
SECISBP2	S1PR5					
SEMA7A	SCYL1					
SESN2	SDHAF2					
SF3B1	SEC24C					
SHKBP1	SELRC1					
SHMT1	SENP1					
SKAP1	SERINC2					
SLC16A6	SEZ6L2					
SLC6A10P	SHMT1					
SLC6A20	SIPA1					
SORT1	SLC18A3					
SOX5	SLC1A2					
SPAG9	SLC24A6					
SPATA8	SLC2A1					
SPOCK2	SLC30A3					
SRGAP2	SLC35F5					

SRSF5	SLC46A1						
SSBP2	SMARCD1						
STC1	SMARCE1						
STT3B	SMCR7						
SYNC	SMOC1						
TAF5	SNAPIN						
TMEM132E	SPAST						
TMEM27	SPATA6						
TMEM62	SRSF2						
TMEM80	ST6GALNAC3						
TMEM97	STARD13						
TNNI1	STMN1						
TNPO3	SUV39H2						
TOB1	SYNRG						
TOR1AIP1	SYT9						
TOX2	TAGLN						
TP53I13	TAGLN2						
TPI1P2	TBCB						
TRIM2	TCF4						
TSC22D1	TEAD2						
TUG1	TGFB1						
UBE2H	TIMELESS						
UBE2R2	TIPRL						
UBE3A	TLX2						
USP32	TMED10						
USP5	TMEM150A						
VAPA	TMUB2						
VAV1	TRIM28						
VCPIP1	UBE2O						
WDR81	UGP2						
WIPI1	ULK1						
WNT3	UPF2						
WNT5A	VAMP2						
XPO1	VASP						
YWHAE	WDR81						
YWHAQ	WNT2B						
ZBTB26	XPR1						
ZBTB32	ZNF48						
ZBTB47	ZNF524						
ZMYM2	ZNF768						
ZMYND8							
ZNF148							
ZNF362							
ZNF367							
ZNF462							
ZNF800							
ZSWIM2							
